# Enhanced disease resistance in chickpea and canola via MAGPIE: Major‐Effect Allele and Genomic Polygenic Integration for Enhancement

**DOI:** 10.1002/tpg2.70326

**Published:** 2026-10-12

**Authors:** Zibei Lin, Irene van den Berg, Shahin Yazdifar, Shimna Sudheesh, Adnan Riaz, Denise Barbulescu, Yongjun Li, Kristy Hobson, Muhammad Ahsan Asif, Matthew Hayden, Sukhjiwan Kaur

**Affiliations:** ^1^ Agriculture Science and Technology, Department of Energy Environment and Climate Action, AgriBio, Centre for AgriBioscience Bundoora Victoria Australia; ^2^ School of Applied Systems Biology La Trobe University Bundoora Victoria Australia; ^3^ NSW Department of Primary Industries and Regional Development Tamworth New South Wales Australia

## Abstract

The global agricultural industry suffers major economic losses due to fungal diseases. Though selective breeding and introgression of resistance genes with major effects have helped to reduce the impact of these diseases, resistance breakdown occurs within a few generations. Quantitative resistance that relies on multiple small effect genes has proven to be more durable than relying on major effect genes. In this study, we present MAGPIE (Major‐Effect Allele and Genomic Polygenic Integration for Enhancement), a computational framework that integrates genomic data, predictive breeding approaches, and cross selection to accumulate favorable major‐effect alleles and polygenic resistance. MAGPIE was implemented to improve fungal resistance in chickpea and canola. Seven cycles of selection resulted in 76% genetic gain for resistance to Ascochyta blight in chickpea, and 15% genetic gain for blackleg resistance in canola. For both diseases, more than 90% of genetic gain could be attributed to polygenic resistance rather than major effect genes. Progenies from both breeding programs were field validated, with stem breakage being reduced by 9.7% in chickpea, and survival percentage improved by 10% compared to the founder parents in canola. These results highlight the power of integrating genomic data and predictive breeding approaches to deliver durable, polygenic resistance. This work demonstrates a scalable framework for accelerating resistance improvement in crops vulnerable to rapidly evolving fungal pathogens.

## Plain Language Summary

Fungal diseases cause major losses in crop production worldwide and can quickly overcome disease resistance developed through conventional breeding. Improving long‐lasting resistance is therefore a major challenge for crop breeders. In this study, we tested whether modern breeding approaches could accelerate the development of disease‐resistant chickpea and canola lines. After seven speed breeding cycles completed within 3 years, resistance to fungal disease improved substantially in both crops. New chickpea lines from early cycles already had 9.7% less stem damage from disease, while new canola lines from early cycles had 10% higher plant survival than the original parental lines. Most of the improvement came from combining many small‐effect resistance genes rather than relying on major resistance genes. These findings show that modern breeding strategies can help deliver stronger and potentially more durable disease resistance in crops.

**Abbreviations**
AB: Ascochyta blight
AveIntInf: average internal infection
BLUEs: best linear unbiased estimates
GBLUP: genomic best linear unbiased prediction
GEBV: genomic estimated breeding value
G × E: genotype‐by‐environment interactions
LD: linkage disequilibrium
MAGPIE: Major‐Effect Allele and Genomic Polygenic Integration for Enhancement
OCS: optimal contribution selection
OHV: optimal haploid value
PCA: principal component analysis
PIP: posterior inclusion probability
QTL: quantitative trait loci
R gene: resistance gene with major effect
SNP: single nucleotide polymorphism
SSD: single seed descent
SurPct: survival percentage

## 1 INTRODUCTION

Fungal diseases result in major economic losses to the global agricultural industry (Fisher et al., 2012; Steinberg & Gurr, 2020; Stukenbrock & Gurr, 2023). Climate change could worsen these losses, as rising temperatures may allow fungi to thrive in areas where they previously could not survive (Bebber et al., 2013; Chaloner et al., 2021). Fungi are fast evolving pathogens, which making it challenging to breed for durable resistance (Möller & Stukenbrock, 2017; Stukenbrock & Gurr, 2023). Selective breeding and introgression of resistance genes with major effects (R genes) have helped the agricultural industry to reduce the impact of fungal diseases. However, widespread use of R gene‐carrying varieties and limited genetic diversity increase selection pressure, leading to resistance breakdown within a few generations (Maurya et al., 2021). An alternative strategy to the selection for R genes is the utilization of quantitative resistance (Maurya et al., 2021; Nelson et al., 2018), which is achieved through the cumulative effects of multiple genes with small effects. If this quantitative resistance is achieved through multiple genes working independently of each other, the pathogen will need to overcome all these resistance mechanisms to infect the plant. Consequently, quantitative resistance may be more durable than qualitative resistance relying on fewer R genes with major effects.

Ascochyta blight (AB) is a major fungal disease affecting chickpea production globally and can cause yield losses up to 100% depending on environmental conditions (Singh et al., 2022; Udupa & Baum, 2003). Improving AB resistance has been a key focus of chickpea breeding programs worldwide (Luthra et al., 1941; Pande et al., 2005), including in Australia, where the industry was only established in the late 1970s (Siddique et al., 2000). Despite significant breeding efforts, resistance breakdowns have been a recurring challenge. The first major breakdown of AB resistance in Australia occurred in the late 1990s when new virulent pathogen strains rendered all existing varieties susceptible. The release of PBA HatTrick in 2009 marked a significant advancement in AB resistance. However, its resistance was compromised within a few years, with the emergence of new aggressive isolates was reported in field in 2016. Subsequently, PBA Seamer, introduced in 2016 with enhanced resistance, also exhibited rapid breakdown under field conditions. Since then, the percentage of highly aggressive isolates has risen significantly. For example, the percentage of isolates that are highly aggressive on PBA Seamer has risen from 4% in 2016, to over 90% in 2022 (Bar et al., 2025). AB resistance is a highly heritable trait (Labdi et al., 2015; Lichtenzveig et al., 2002; Lin et al., 2025). Though several R genes (Dey & Singh, 1993; Labdi et al., 2013) and quantitative trait loci (QTL) have been identified (Daba et al., 2016; Deokar et al., 2019; Lin et al., 2025), a previous study showed that 67% of the genetic variance in AB resistance was attributed to polygenic variance, that is, quantitative resistance (Lin et al., 2025), and none of the major genes identified to date offer resistance against the new aggressive isolates. The rapid evolution of the AB pathogen continues to outpace breeding efforts (Ilyas et al., 2022), and currently, all chickpea varieties in Australia are susceptible to the disease (GRDC, 2025). This highlights the ongoing and urgent need to find new sources of resistance and develop more durable resistance against new aggressive isolates.

In another case, a major fungal disease affecting canola is blackleg, caused by *Leptosphaeria maculans*, which results in substantial yield losses in Australia, Canada, and Europe (West et al., 2001). Average annual yield losses of between 10% and 20% have been reported for Australia, Canada, and the United Kingdom (Van de Wouw et al., 2016, 2021; Zhang & Fernando, 2017). However, individual losses can be much larger, with yield losses of up to 90% reported in Australia (Van de Wouw et al., 2021). Various R genes have been detected and deployed in canola breeding (Vasquez‐Teuber et al., 2024), including *Rlm1* (Zhai et al., 2021), *Rlm2* (Larkan et al., 2015), *Rlm3* (Delourme et al., 2004), *Rlm4* (Tollenaere et al., 2012), *Rlm7* (Delourme et al., 2004), *Rlm9* (Larkan et al., 2020), *LepR1* (Yu et al., 2005), *LepR2* (Yu et al., 2005), *LepR3* (Larkan et al., 2013), *LepR4* (Yu et al., 2013), *BLMR1* (Long et al., 2011), and *BLMR2* (Long et al., 2011). However, similar to AB in chickpea, the introduction of R genes in canola blackleg is often followed by fast resistance breakdown (Rouxel et al., 2003; Van de Wouw et al., 2021; Zhang et al., 2016). In Australia, resistance breakdown occurred within 3 years of the release of commercial varieties carrying major gene *LepR3* in 2002 (Sprague et al., 2006). Similarly, due to the breakdown of resistance to *Rlm1* in 2011, Australian farmers had to switch to varieties that carried different resistance genes to avoid major yield losses in 2012 (Van de Wouw et al., 2014). In Canada, widespread use of the *Rml3* resistance gene has likely contributed to *Rml3* resistance breakdown (Zhang et al., 2016). Consequently, it has been suggested that quantitative resistance can help to increase the durability of resistance to blackleg pathogen (Amas et al., 2021; Brun et al., 2010). A previous study reported that the percentage of genetic variance explained by QTL regions for survival and internal infection ranged from 2% to 33% (Fikere et al., 2018). Hence, like AB resistance in chickpea, the majority of genetic variance for blackleg resistance can be explained by polygenic variance rather than R genes or QTL.

Given the rapid breakdown of major‐gene resistance and the polygenic nature of fungal resistance, there is a clear need for breeding strategies capable of efficiently capturing this polygenic variance to deliver durable resistance. Genomic selection is widely used in plant breeding (Alemu et al., 2024; Kumar et al., 2024), to improve simple to complex traits including disease resistance (Bekele et al., 2019; Poland & Rutkoski, 2016), as it can capture both major‐gene and polygenic sources of resistance simultaneously. To further accelerate genetic gain, genomic selection can be integrated with speed breeding techniques and crossing designs. Speed breeding significantly reduces generation intervals, which increases the number of breeding cycles per year, expediting the development of improved germplasm (Watson et al., 2018). Parental/cross combination selection based on genomic estimated breeding values (GEBVs) or optimal haploid values (OHVs) (Daetwyler et al., 2015; Kemper et al., 2012) enables the accumulation of favorable alleles, maximizing genetic gain. For instance, a pool of 100 candidates can result in a total of 4950 potential cross combinations, while only a small number of combinations (e.g., *n* = 50) can be tested in practice due to constraints of resources. Conventionally, cross selection strategies have heavily relied on breeders’ knowledge and experience which, though valuable, can be subjective and may overlook potentially superior combinations. To address this limitation, our study introduces a novel simulation‐based method, MAGPIE (Major‐Effect Allele and Genomic Polygenic Integration for Enhancement), to systematically and objectively identify the most promising parent combinations. By integrating genomic data, predictive models, and inbreeding restrictions, MAGPIE enables breeders to prioritize crosses that are expected to deliver the greatest genetic gain, thereby optimizing resource allocation and accelerating breeding progress. The objective of this study was to validate the efficacy of MAGPIE combined with speed breeding in improving AB resistance in chickpea and blackleg resistance in canola.

## 2 MATERIALS AND METHODS

In this paper, MAGPIE was developed and implemented to enhance disease resistance for AB in chickpea and blackleg in canola, serving as case studies for its application. An overview of the MAGPIE workflow used in this study is provided in Figure 1. Briefly, genomic prediction models were trained using phenotypic and genotypic data and integrated into MAGPIE for predicting the performance of simulated progenies from all possible parental combinations. Subsequently, the selected cross combinations from MAGPIE were generational advanced in speed breeding and phenotypic validation to accelerate genetic gain for disease resistance.

**FIGURE 1:**
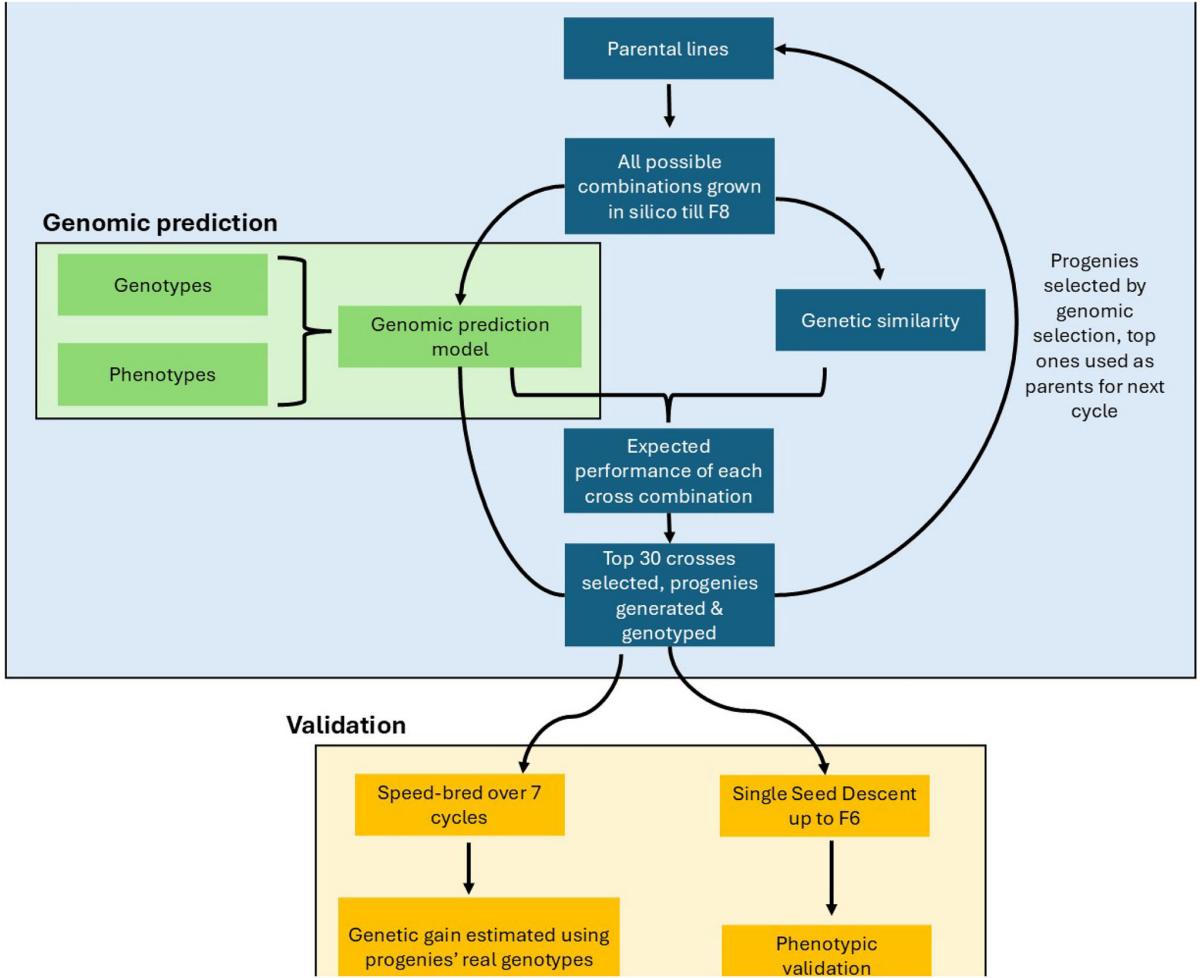
Overview of the MAGPIE (Major‐Effect Allele and Genomic Polygenic Integration for Enhancement) workflow. Phenotypic and genotypic data were used to develop genomic prediction models. These models were applied to simulated progeny from all possible parental combinations to estimate expected cross performance. Crosses predicted to generate superior progeny were selected for subsequent crossing cycles, while selected progeny were advanced through single seed descent and phenotypically validated to confirm realized genetic gain.

### 2.1 Phenotypes and genotypes for genomic prediction model training

#### 2.1.1 Chickpea AB

Details of phenotypes, genotypes, and training of the genomic prediction model for chickpea AB were previously published in Lin et al. (2025). In brief, a total of 2790 chickpea lines were provided by the Australian Grains Genebank, Horsham, Australia, which were originally collected from multiple sources, including advanced breeding material, wild *Cicer* introgression and commercial varieties. Phenotyping of the 2790 lines was conducted over3 years (2020–2022) using both field trials and an outdoor pot‐based assay, with stem breakage percentage (1%–100%) recorded as the key trait for assessing resistance to AB. Best linear unbiased estimates (BLUEs) were estimated across field trials and pot‐based assays. In addition, strong genetic correlations (ranging from 0.7 to 0.9) were estimated between field trials in different years and between pot‐based assays, indicating limited genotype‐by‐environment interactions (G × E) in the chickpea AB study (Lin et al., 2025). All lines were genotyped using Illumina Multispecies Pulse 30K single nucleotide polymorphism (SNP) Array and imputed to a whole‐genome sequence level with 765,308 SNPs, followed by stringent filtering based on a linkage disequilibrium (LD) sliding window size of 200, *R* ^2^ of pairwise LD *< *0.99, *R* ^2^ of imputation *> *0.8, and minor allele frequency *> *0.05. After these filters, a total of 23,239 high‐quality genome‐wide SNPs were obtained for genomic prediction. Multiple cross‐validation scenarios were evaluated to determine whether disease resistance could be accurately predicted from maker data. The genomic best linear unbiased prediction (GBLUP) model (VanRaden, 2008) achieved an average prediction accuracy of 0.72, indicating that genomic predictions were sufficiently reliable to support selection decisions. The optimized prediction model was subsequently used within MAGPIE to identify parent combinations predicted to generate the most disease‐resistant progenies.

#### 2.1.2 Canola blackleg

To develop genomic prediction models for blackleg resistance, phenotypic data were collected from six field trials, conducted in 2015 (2 trials), 2018 (2 trials), 2021 (1 trial), and 2023 (1 trial) at three locations in Victoria, Australia (Table 1). The 2015, 2018, and 2021 trials have been described in detail by Fikere et al. (2018) and Zhao et al. (2025). Briefly, the field trials conducted in 2015 were at Green Lake and Lake Bolac, and evaluated 597 spring and winter canola lines. The spring lines were Australian commercial cultivars and Agriculture Victoria breeding germplasm and the winter lines were sourced from the Australian Grains Genebank. All later trials were conducted at Wonwondah. In 2018, one field trial consisted of 1121 OHV doubled haploid lines, and a second trial consisted of 215 spring lines. The 2021 and 2023 trials consisted of 401 and 577 lines, respectively. The lines used in the 2021 and 2023 trials were a combination of spring lines, OHV doubled haploids and speed breeding lines. In each trial, blackleg resistance was assessed using two complementary traits: survival percentage (SurPct) and average internal infection (AveIntInf). SurPct was calculated as $\mathrm{SurPct}\;=\frac{\mathrm{nSurv}}{\mathrm{nEme}}\times 100\%$, where nSurv equals the surviving plant count at maturity, and nEme the number of plants emerged 6 weeks after sowing. AveIntInf is the percentage of blackleg impact area of the total stem cross‐section. BLUEs were estimated using ASReml‐R (Butler et al., 2009) to account for spatial variation within trials. Previously estimated genetic correlations between trials for AveIntInf and SurPct were high, indicating limited G × E for blackleg resistance in canola (Zhao et al., 2025). Therefore, within trial BLUEs were corrected for trial effect, resulting in the overall BLUEs that were used as phenotypes for all subsequent analysis.

**TABLE 1:** Descriptive statistics of blackleg trials. Mean and standard deviation of BLUEs estimated per trial are provided for average internal infection (AveIntInf) and survival percentage (SurPct).

Year	Location	nLines	Description	AveIntInf	SurPct
2015	Green Lake	597	Spring and winter lines	57.1 ± 16.9	56.1 ± 15.8
2015	Lake Bolac	597	Spring and winter lines	83.8 ± 13.2	22.9 ± 17.8
2018	Wonwondah	1121	Doubled haploids	63.3 ± 17.1	55.5 ± 20.6
2018	Wonwondah	215	Spring lines	69.6 ± 13.5	50.4 ± 17.3
2021	Wonwondah	401	Spring lines and doubled haploids	72.2 ± 14.5	41.0 ± 17.7
2023	Wonwondah	577	Spring lines, doubled haploids, and speed breeding lines	77.7 ± 11.6	32.1 ± 15.4

The genotyping pipeline and QC for the canola dataset has been previously described by Zhao et al. (2025). In brief, individuals were genotyped with a mix of genotyping technologies, including GBS‐t, skim‐WGS, and WGS at 10x coverage (Malmberg, Barbulescu, et al., 2018; Malmberg, Shi, et al., 2018). To create a single, consistent marker dataset across all lines, GBS‐t and skim‐WGS were imputed to WGS. GBS‐t was imputed using Eagle (Loh et al., 2016) for phasing and Minimac3 (Das et al., 2016) for imputation, while skim‐WGS was imputed using GLIMPSE (Rubinacci et al., 2021). After merging the datasets, 48,732 SNPs and 2629 individuals were retained for further analysis. Because the dataset consisted of both winter and spring canola lines, population structure was investigated using a principal component analysis (PCA). The PCA was performed in GCTA using a genomic relationship matrix calculated according to Yang et al. (2011). Genomic prediction was performed using BayesR3 (Breen et al., 2022), fitting the first principal component in the PCA as covariate to account for population structure. BayesR3 is a computationally efficient implementation of a Bayesian variable selection model in which marker effects are assigned to one of four distributions with increasing variances, including a zero‐effect distribution (Erbe et al., 2012). Five Monte Carlo Markov Chains of 50,000 iterations were run, with 40,000 burn‐in iterations. Genomic prediction accuracy for AveIntInf and SurPct was assessed using fivefold random cross‐validation, where lines in the test set were predicted using models trained on the remaining lines. This process was repeated 10 times. Prediction accuracy was estimated as the Pearson correlation between predicted GEBVs and overall BLUEs. Once prediction accuracy was established, BayesR3 (Breen et al., 2022) was used to predict GEBVs for all candidate parents and guide cross selection within MAGPIE.

#### 2.1.3 MAGPIE—Advanced in silico approach

MAGPIE was used to identify the parent combinations most likely to produce superior progeny in the next breeding cycle. Rather than generating and testing thousands of crosses experimentally, MAGPIE evaluates potential crosses using computer simulations, allowing breeding resources to be focused on the most promising parent combinations. Initially, candidates were selected via the optimized genomic prediction models as described above, where real genotypes of the selected candidates were utilized as input for MAGPIE. All possible cross combinations of the selected candidates were simulated using a computer program (*CropSim*, coded in c++) developed by Agriculture Victoria Research (Lin et al., 2016). Genotypes were then grown in silico from the parental lines through to F_8_ stage, which approximates the level of homozygosity typically targeted for breeder‐ready lines. For each possible combination, an F_1_ population was simulated and advanced through single seed descent (SSD) from F_2_ to F_8_. Twenty progenies were simulated per cross to capture within family variance. Genomic prediction models described above were then used to predict the GEBVs of the simulated progenies. The expected performance of each cross combination was calculated as the average GEBV of its simulated F_8_ progeny. To reduce stochastic variation, the entire simulation process was repeated 20 times and results were averaged across replicates.

To maintain genetic diversity and reduce the risk of inbreeding, genetic similarity was estimated by counting the number of SNP alleles in common for every pair of parents, where combinations from a pair of parents with >90% SNP alleles in common were excluded. The remaining cross combinations were ranked according to their predicted progeny performance, and the top 30 crosses were selected as parents for next crossing cycle sown in speed breeding glass house.

In parallel, F_1_ of the selected crosses from each alternate crossing cycle were advanced up to F_6_ generation via SSD in the speed breeding glass house, to generate seed stocks for field validation trials. The overall MAGPIE workflow therefore combined genomic prediction, computer simulation, and rapid generation advancement to accelerate genetic gain.

The same approach was used for both diseases (i.e., chickpea AB and canola blackleg). In chickpea, selection decisions were based on stem breakage GEBVs. In canola, selections were based on a desired gain index (Itoh & Yamada, 1986) that combined blackleg resistance traits (AveIntInf and SurPct) with other breeding objectives (yield, vigor score, oil content, glucosinolate content, protein content, and oleic acid).

#### 2.1.4 Validation trials

To evaluate whether the genetic gains predicted by MAGPIE translated into real improvements in disease resistance, the selected progenies were advanced for phenotypic evaluations. Lines from every second speed‐breeding cycle were advanced to F_5_ or F_6_ through SSD, depending on seed availability at the time of evaluation. This breeder‐ready germplasm was subsequently used in validation trials to test whether the gains predicted by MAGPIE were realized phenotypically.

The chickpea AB validation was conducted under controlled conditions in a temperature controlled polytunnel by Chickpea Breeding Australia in 2024. MAGPIE‐selected lines that had been generationally advanced via SSD from speed breeding cycles 1 (F_5_) and 3 (F_6_), plus the 52 founder parents from the diversity panel, were evaluated for AB resistance. Twelve genotypes with five seeds per genotype were sown in a 25 L (580L × 385 W × 166H mm) vented tub. After sowing, plants were grown and maintained under natural light at temperature 25 ± 2°C. At the three‐node stage, approximately 12–16 days after sowing, plants were inoculated with a spore suspension (500,000 spores m^−1^) of mix isolates using a Nylex 8L shoulder sprayer until runoff. The isolate mixture included five highly aggressive (TR10876, TR10872, TR10900, TR9668, and TR9571) and one relatively less aggressive isolate (TR9126), enabling resistance to be assessed against a diverse pathogen population. Temperature was maintained at 20°C after inoculation. Seedlings were misted for 30 s every 15 min for 24 h after inoculation. Twenty‐four hours later, watering level was changed to 5 min once or twice per day, depending on weather conditions, until disease assessment. Disease severity was recorded 14–18 days after inoculation by counting the plants having stem breakage and converted to percentage stem breakage.

The 2023 canola blackleg trial at Wonwondah (described above) included both the founder parents and SSD lines derived from cycles 1 and 3 of the MAGPIE pipeline. This provided an opportunity to directly compare the disease resistance of the original breeding material with that of progeny generated through MAGPIE. Within trial BLUEs for AveIntInf and SurPct were used to assess whether the predicted genetic gains translated into improved blackleg resistance under field conditions.

#### 2.1.5 Data analysis

To determine whether the genetic gains achieved through MAGPIE were primarily driven by major gene or the cumulative effects of many small‐effect variants, QTL were identified within the same training populations that were used to develop the genomic prediction models. For chickpea AB resistance, QTL were defined as the markers that explained >1% genetic variance estimated by BayesR3 modelling (Lin et al., 2025). For blackleg resistance in canola, variants with a posterior inclusion probability (PIP) ≥ 0.4 for AveIntInf and SurPct were considered QTL. BayesR3 assigns each marker to one of four distributions corresponding to no, small, medium effect, or large effects (Breen et al., 2022). The PIP was calculated as 1 minus the posterior inclusion probability that a marker had no effect. Consequently, PIP represents the probability that a marker contributes to genetic variation for the trait, with larger PIP values providing stronger evidence that a marker is associated with the phenotype. Markers exceeding the PIP threshold were classified as QTL, while all remaining markers were considered part of the polygenic variance. To partition the sources of genetic gain, GEBVs were subsequently estimated using either QTL markers (GEBV_QTL) or at remaining markers (GEBV_poly), allowing the relative contributions of major loci and polygenic variation to be quantified.

The change in the rate of genetic gains over cycles were quantified by the percentage of GEBV increased between the founder parents and the MAGPIE‐selected lines generated in speed breeding crossing cycles as $\frac{\mathrm{mean}\left(\mathrm{GEB}V_{\mathrm{cycle}\_N}\right)\;-\;\mathrm{mean}\left(\mathrm{GEB}V_{\mathrm{founder}\_\mathrm{parents}}\right)}{\mathrm{mean}(\mathrm{GEB}V_{\mathrm{founder}\_\mathrm{parents}})}\times 100\%$, where $\mathrm{mean}(\mathrm{GEB}V_{\mathrm{cycle}\_N})$ is the mean GEBV of lines in speed breeding cycles, and N ∈ 1, 2, …7, and $\mathrm{mean}(\mathrm{GEB}V_{\mathrm{founder}\_\mathrm{parents}})$ is the mean GEBV of the founder parents selected at the commencement of projects. This metric provides a measure of the response to selection and tracks changes in resistance across breeding cycles. For AveIntInf, where an increased breeding value means reduced disease resistance, the genetic gain was multiplied by −1 to make it comparable with other traits, so that a positive genetic gain means improved disease resistance. In addition, phenotypes from validation trials were also summarized for the founder parents and the speed‐bred SSD progenies of cycles 1 and 3 via box plot using “ggplot2” of R‐library (Wickham, 2016). These comparisons were used to determine whether the predicted genetic gains were reflected in observed disease resistance. In addition, to assess the genetic diversity among lines generated in each cycle, the expected heterozygosity for each SNP was calculated within each cycle following Nei (1973), as $H_{e}=2p\left(1-p\right)$, where *p* denotes the frequency of the reference allele.

To provide an indication of the potential phenotypic response in later breeding cycles, the expected stem‐breakage performance of cycle 7 chickpea lines was projected from the relationship between realized phenotypic improvement and genetic gain observed in the validation cycles. Specifically, the reduction in stem breakage observed between the founder parents and SSD progeny from cycles 1 and 3 was compared with the corresponding increase in GEBVs over the same interval. The resulting ratio was then used to estimate the expected phenotypic response associated with additional genetic gain. This projection assumed that the relationship between genetic gain and realized phenotypic improvement remains approximately linear across breeding cycles and was used solely to provide an indicative estimate of cycle 7 performance.

## 3 RESULTS

Significant genetic gain for resistance to both fungal diseases was achieved through MAGPIE, and phenotypic validation demonstrated that these gains translated into improved disease resistance in the field.

### 3.1 AB resistance in chickpea

The crossing and selection strategy implemented through MAGPIE resulted in progressive improvements in AB resistance across successive cycles. When compared to the founder parents, those selected in subsequent MAGPIE cycles consistently achieved greater genetic gains, namely, more disease resistance (Figure 2). Genetic gains increased from 10% in cycle 1, to 75.7% in cycle 7, of which the majority was contributed by the quantitative SNPs. As shown, approximately 90% of the genetic gain was derived from these quantitative SNPs, while the remaining gain was contributed by major QTL.

**FIGURE 2:**
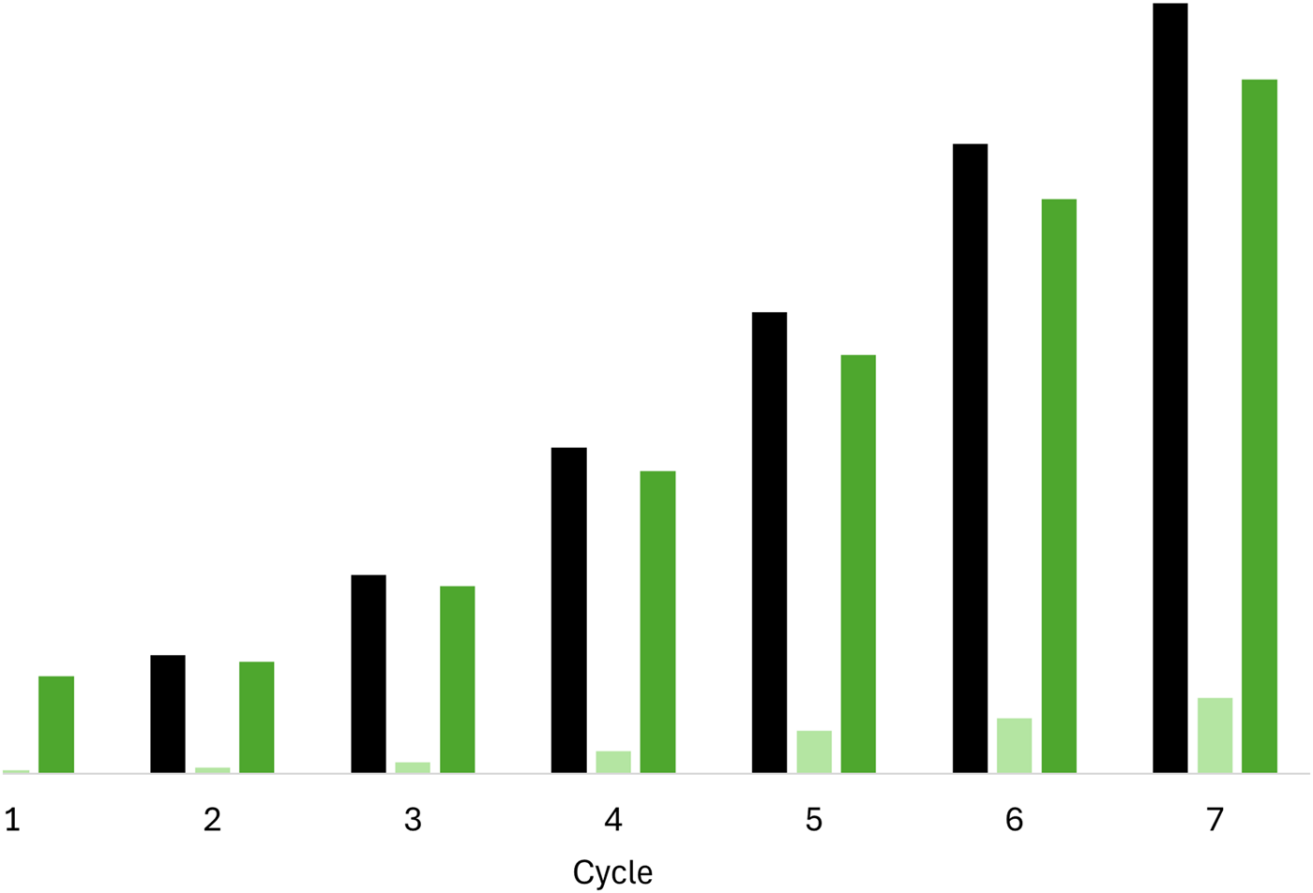
Genetic gain of Ascochyta blight resistance across seven Major‐Effect Allele and Genomic Polygenic Integration for Enhancement (MAGPIE) cycles in chickpea. The x‐axis shows the MAGPIE cycle, and the y‐axis shows the genetic gain (%) relative to the founder parents based on genomic estimated breeding values. Total genetic gain (total) was partitioned into gain attributed to major quantitative trait loci (QTL) and polygenic marker effects (polygenic). Genetic gain increased from 10% in cycle 1 to 75.7% in cycle 7, with approximately 90% of the gain attributable to polygenic effects. The QTL were identified in Lin et al. (2025).

In the chickpea validation study, founder parents displayed a mean of 54.33% stem breakage, compared to 47.69% and 44.62% observed from the progenies of cycles 1 and 3, respectively (Figure 3). Compared to the top 10 founder parents, the best 10 lines advanced through SSD from cycle 3 exhibited significantly lower stem breakage scores (averaged BLUEs 7.7 vs. −2.9, respectively). It is worth noting that 21 SSD lines developed in this study were found having no breakage, in other words, completely resistant to the AB isolates applied in the validation trial. Furthermore, based on predicted genetic gains and validated phenotypic data, each 1% increase in genetic gain corresponded to approximately a 0.32% reduction in stem breakage. Founder parents exhibited 54.33% stem breakage, compared with 47.69% and 44.62% for SSD progeny from cycles 1 and 3, corresponding to reductions of 6.64% and 9.71%, respectively (Figure 3). Over the same interval, genetic gain increased from 10.0% in cycle 1 to 19.5% in cycle 3 (Figure 2). This relationship equated to an approximate 0.32% reduction in stem breakage for every 1% increase in genetic gain. Assuming this relationship remained constant across cycles, the genetic gain achieved in cycle 7 (75.7%) would correspond to an expected average stem breakage of 26.66%.

**FIGURE 3:**
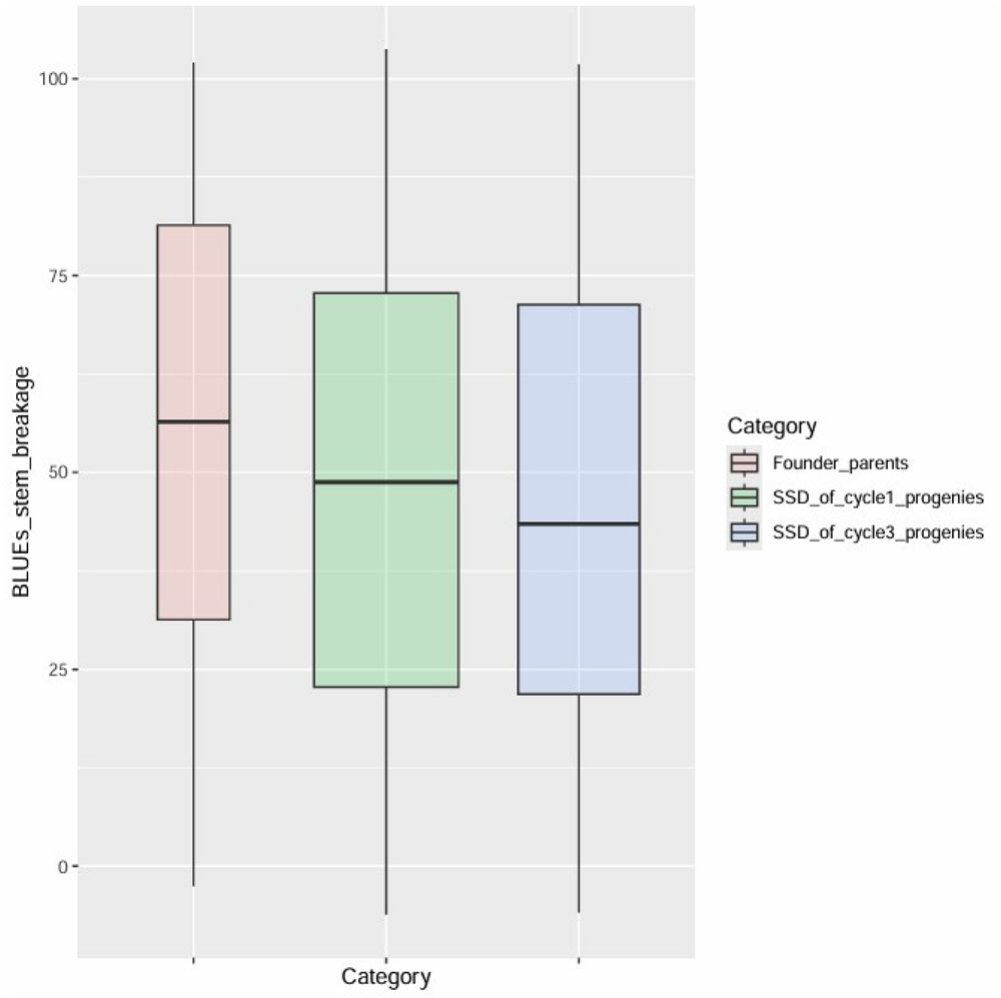
Phenotypic validation of Ascochyta blight (AB) resistance in chickpea founder parents and single seed descent (SSD) progeny derived from MAGPIE (Major‐Effect Allele and Genomic Polygenic Integration for Enhancement) cycles 1 and 3. The y‐axis shows the best linear unbiased estimates (BLUEs) for stem breakage following inoculation with AB isolates, where lower values indicate greater resistance. Boxplots represent the distribution of stem breakage scores for founder parents and progeny from cycles 1 and 3. Stem breakage was lower in progeny derived from MAGPIE cycles 1 and 3 than in the founder parents, indicating improved resistance.

Expected heterozygosity was calculated for each cycle based on the available genotype data (Table S1). Overall, expected heterozygosity decreased gradually across cycles (from 0.22 in cycle 1 to 0.17 in cycle 7), reflecting a reduction in genetic diversity due to selection pressure.

### 3.2 Blackleg resistance in canola

Genomic prediction accuracies for blackleg resistance in canola were 0.687 for AveIntInf and 0.695 for SurPct. Genetic gain for both SurPct and AveIntInf fluctuated during the first five cycles (Figure 4), when maintaining genetic diversity was a major selection factor. From cycle 6 onward, consistent improvements in genetic gain were made, with a 15% increased SurPct and 11% reduction in AveIntInf observed in cycle 7 compared to the founder parents. The genetic gain for both SurPct and AveIntInf was driven by polygenic variance rather than variance at QTL. For example, the genetic gain in SurPct observed in cycle 7 compared to founder parents (+14.9%) was largely due to an increase in the GEBV at polygenic variants (+13.7%), while the contribution of QTL was minimal (+1.2%). Figure S1 visualizes the QTL detected for SurPct and AveIntInf.

**FIGURE 4:**
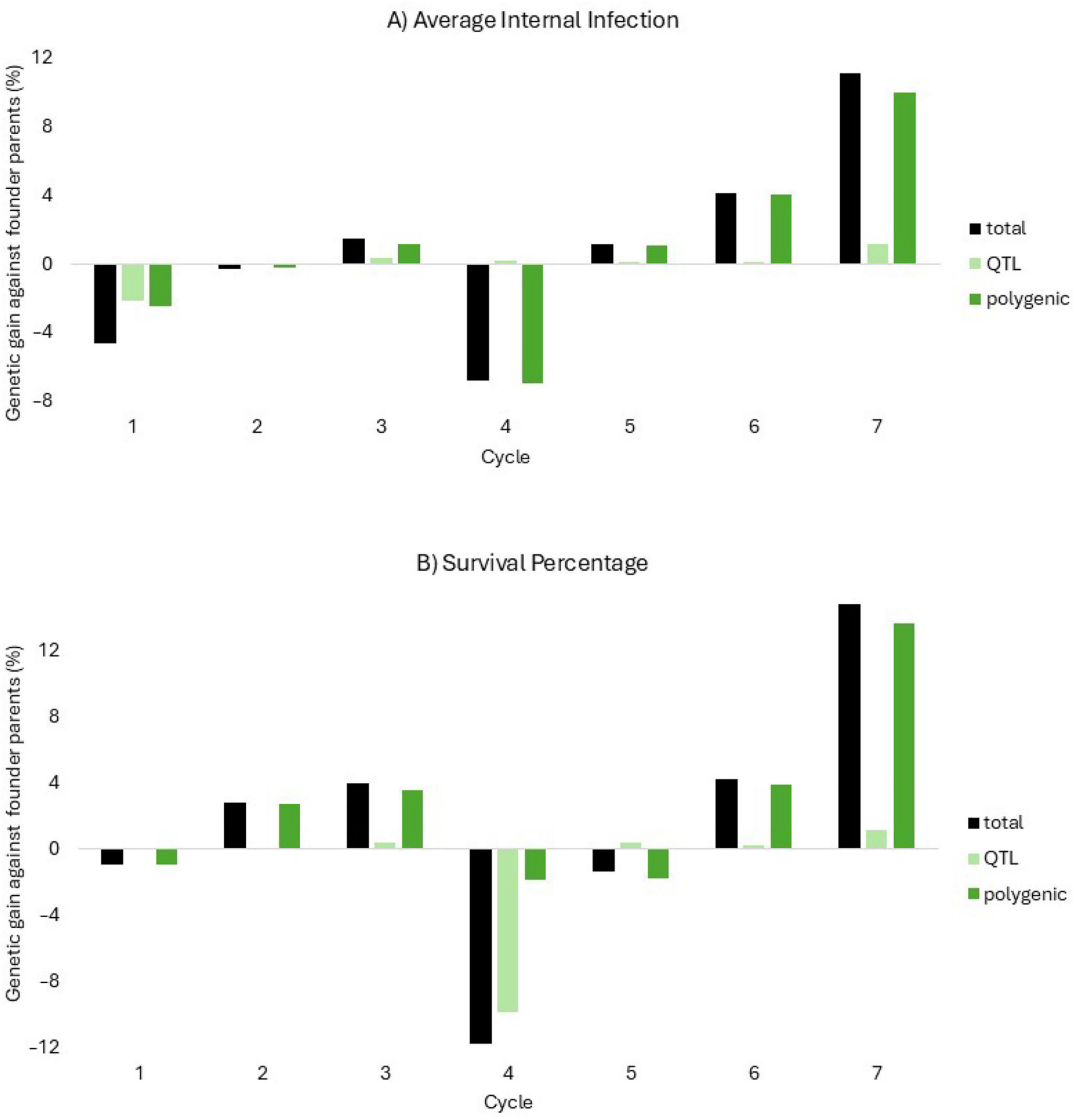
Genetic gain of blackleg resistance across seven MAGPIE cycles in canola. The x‐axis represents the MAGPIE (Major‐Effect Allele and Genomic Polygenic Integration for Enhancement) cycle and the y‐axis the genetic gain (%) relative to the founder parents based on genomic estimated breeding values. Blackleg resistance was measured as average internal infection (A) and survival percentage (B). Total genetic gain (total) was partitioned into gain attributed to major quantitative trait loci (QTL) and polygenic marker effects (polygenic). Genetic gain varied among early cycles but increased to 11% for average internal infection and 15% for survival percentage by cycle 7, with approximately 90% of genetic gain attributable to polygenic effects.

Genetic gains were confirmed by the canola validation study, where MAGPIE resulted in improved disease resistance for both traits (Figure 5). AveIntInf was reduced to 72% in the cycle 3 SSD progeny, compared to 81% for the founder parents. A similar trend was observed for SurPct, which equaled 29% in the founder parents and increased up to 39% in the SSD of cycle 3 progeny. Larger improvement in disease resistance was achieved for the 10 lines with highest disease resistance (highest SurPct and lowest AveIntInf) than for the 10 lines with lowest disease resistance (Table 2). For example, SurPct was improved to 66.2% in the top 10 of SSD of cycle 3 progeny as compared to 51.9% in the top 10 of founder parents, while no improvement was made in SurPct for the bottom 10 lines. Expected heterozygosity decreased from 0.32 in cycle 1 to 0.26 in cycle 2 and remained relatively stable in later cycles, ranging from 0.23 to 0.25 across cycles 3 to 8 (Table S1).

**FIGURE 5:**
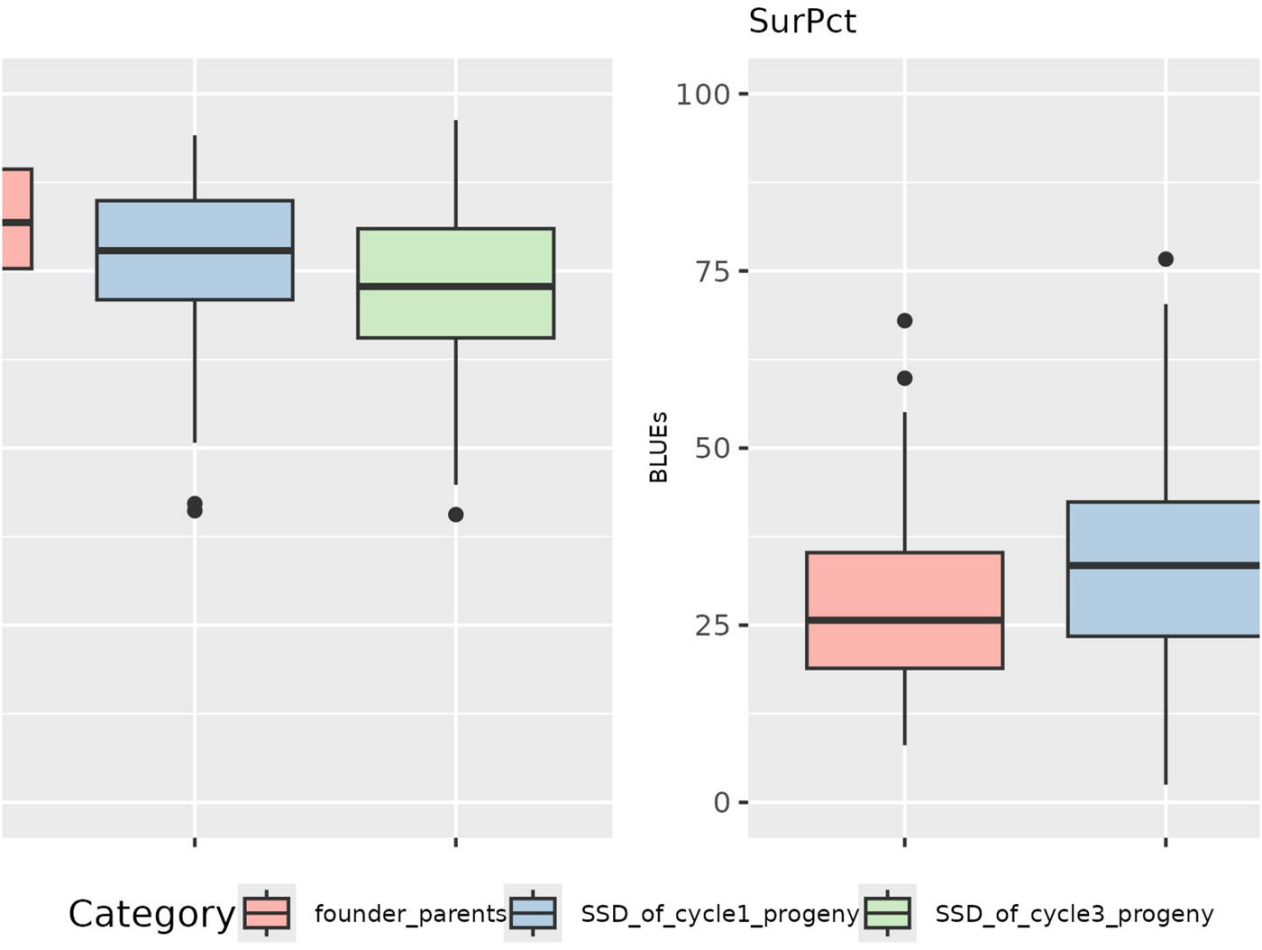
Phenotypic validation of blackleg resistance in canola founder parents and single seed descent (SSD) progeny derived from MAGPIE (Major‐Effect Allele and Genomic Polygenic Integration for Enhancement) cycles 1 and 3. Panel A shows average internal infection (AveIntInf) and panel B shows survival percentage (SurPct). The y‐axis represents the best linear unbiased estimates (BLUEs) for each trait. Lower average internal infection and higher survival percentage indicate greater blackleg resistance. Boxplots show the distribution of trait values for founder parents and SSD progeny from cycles 1 and 3. Compared with the founder parents, progeny from cycles 1 and 3 exhibited reduced internal infection and increased survival.

**TABLE 2:** Average internal infection (AveIntInf) and survival percentage (SurPct) in the 10 best (top10) and 10 worst (bottom10) performing lines in validation study. SSD, single seed descent.

	AveIntInf		SurPct	
	Top10	Bottom10	Top10	Bottom10
Founder_parents	61.8	92.3	51.9	11.4
Cycle 3 SSD progenies	46.9	92.8	66.2	11.4

## 4 DISCUSSION

Our study demonstrates how MAGPIE can rapidly improve resistance to fungal pathogens such as AB in chickpea and blackleg in canola and deliver breeding‐ready germplasm for deployment (e.g., both projects have been conducted for seven crossing cycles, and SSD materials of early cycles were validated within 3 years). In both species, the majority of genetic gain achieved was due to polygenic variance rather than major resistance genes, and polygenic resistance conferred by multiple small effect genes is believed to be more durable than resistance achieved through resistance genes (Maurya et al., 2021; Nelson et al., 2018). Hence, the improved disease resistance achieved through MAGPIE is likely to be more durable than resistance achieved through the introgression of large effect resistance genes.

### 4.1 Genetic gain estimated using progenies’ real genotypes

In chickpea, GEBVs consistently improved across 7 selection cycles, and several lines had complete resistance to the AB isolates applied in the validation trial. As shown in Figure 2, most of the genetic gains achieved by the progenies were attributed to polygenic SNP effects (c. 90%), while QTL contributed to the remaining gain. Compared to conventional breeding strategies focused on breeding for major resistance genes, our study supports a novel approach to breed for durable resistance to multiple AB isolates by accounting for quantitative and QTL effects simultaneously. In addition, selection pressure inevitably resulted in a reduction in genetic variance across selection cycles, as reflected by the decline in expected heterozygosity. However, the implementation of a constraint within the MAGPIE that prevented the mating of highly genetically similar individuals, enabled the retention of genetic diversity in the resulting progeny population which mitigated the potential risk of inbreeding depression.

While MAGPIE improved blackleg resistance in canola, consistent improvements only occurred during the later crossing cycles. During the first five cycles, greater focus was placed on maintaining genetic diversity. Furthermore, selections were not made solely on blackleg resistance, but on a multi‐trait selection index, which likely contributed to these fluctuations. In later cycles, resistance steadily improved in each cycle. While we focused on blackleg resistance in the current study, genetic gain was obtained for all traits in the selection index (unpublished results). In practice, plant breeders need to improve on a range of traits simultaneously, and the canola blackleg case study shows that MAGPIE can result in substantially improved disease resistance while being selected as part of a selection index.

Most of the genetic improvement for blackleg resistance achieved through MAGPIE was driven by gains in polygenic resistance. This finding is consistent with results published in an earlier study (Fikere et al., 2018), where the majority of genetic variance for blackleg resistance was explained by polygenic variance rather than QTL. Similarly, the majority of genetic variance for AB resistance is polygenic variance rather than variance captured by QTL (Lin et al., 2025). MAGPIE can be deployed using a range of genomic prediction models to predict GEBVs. The assumptions underlying genomic prediction models can influence the extent to which GEBVs, and consequently selection decisions, are driven by major resistance genes versus polygenic variation. For example, Bayesian variable selection models (Breen et al., 2022; Habier et al., 2011) allow for some variants to have large effects (i.e., R genes and QTL) while most variants have small or zero effects (i.e., polygenic variance), whereas GBLUP assumes all variants have small effects (VanRaden, 2008) (i.e., polygenic variance). Hence, a genomic prediction model whose prior assumptions match what is known about the biology underlying the trait of interest may be beneficial to maximize benefits obtained from MAGPIE.

### 4.2 Phenotypic validation of progenies’ performance

For both species, phenotypic validation was only available for progeny from cycles 1 and 3. Genomic prediction accuracy may decline across recurrent selection cycles as the selected populations become increasingly divergent from the original training population (Jighly et al., 2019; Müller et al., 2017). Conversely, the availability of additional phenotypic data through the course of the breeding program may improve prediction accuracy by increasing the size of the training population (Edwards et al., 2019). The net effect of these opposing factors is difficult to predict and was not explicitly quantified in the present study. Therefore, the projected stem breakage estimated for cycle 7 in chickpea should be interpreted as an expected response to selection rather than a validated phenotypic outcome. Regular updating of the training population and recalibration of genomic prediction models is important for maintaining long‐term prediction accuracy (Pszczola & Calus, 2016; Sabadin et al., 2022) and realize the full potential of MAGPIE.

### 4.3 Implications for breeding practice

The MAGPIE framework developed in this study was founded on a stochastic optimization approach, with selection criteria that can be tailored to the specific objectives and constraints of individual crop breeding programs. This flexibility makes MAGPIE a versatile and readily implementable framework that can be adapted across crop species and breeding targets. In this paper, progeny populations of both crop species were selected using MAGPIE and subsequently advanced through seven cycles of speed breeding over a 3‐year period under glasshouse conditions and the outcomes have been well received by industry, demonstrating the practical utility of the approach in applied breeding programs. Most published mate selection methodologies are based on deterministic approaches, such as optimal contribution selection (OCS) (Cowling et al., 2023; Meuwissen, 1997), which are generally designed for specific breeding scenarios and objectives. For example, OCS was initially developed for animal breeding, and one of the key requirements is the assignment of each genotype as either male or female. However, in many plant species, individual plants can function as either male or female parents, making such assumptions less practical. In contrast, the MAGPIE approach introduced in our study uses a stochastic framework that models and advances genotypes in silico without imposing gender restrictions. The broader applicability and customizability of MAGPIE may therefore provide advantages in breeding programs with diverse selection goals and operational constraints. Nevertheless, evaluating the potential genetic gain achieved by OCS relative to MAGPIE could be an interesting area for future investigations.

We demonstrated that our MAGPIE pipeline can rapidly improve fungal disease resistance in globally important crop species. While our two case studies focused on disease resistance, the same approach should be able to generalize for any heritable quantitative trait, such as yield, quality, and environmental stress tolerance. The amount of genetic gain will depend on trait dependent factors such as heritability, the genetic architecture of the trait, and the size of the training population. MAGPIE is also applicable to oligogenic traits controlled by a small number of causal loci, if the prediction markers capture the effect of those causal loci. However, for traits controlled by one or a few major‐effect genes, alternative approaches such as marker‐assisted introgression or gene editing may provide a faster route to genetic improvement. In contrast, MAGPIE is likely to provide the greatest advantage for traits controlled by a combination of major and minor loci, or by many minor loci, where favorable alleles must be accumulated across multiple generations. Beyond genetic architecture, the performance of MAGPIE will also depend on the ability of the genomic prediction model to capture other sources of variation such as G × E. In our current implementation, we did not account for G × E, because of the relatively low presence of G × E for the traits we analyzed. However, MAGPIE can be used with any genomic prediction model, including factor analytic models and environmental covariate models, allowing users to account for G × E if required for their traits. Future research will be needed to validate the gains that can be obtained by MAGPIE in a wider range of traits and species.

## AUTHOR CONTRIBUTIONS

**Zibei Lin**: Conceptualization; data curation; formal analysis; investigation; methodology; project administration; software; supervision; validation; visualization; writing—original draft; writing—review and editing. **Irene van den Berg**: Data curation; formal analysis; investigation; validation; visualization; writing—original draft; writing—review and editing. **Shahin Yazdifar**: Methodology; writing—review and editing. **Shimna Sudheesh**: Methodology; writing—review and editing. **Adnan Riaz**: Formal analysis; writing—review and editing. **Denise Barbulescu**: Data curation; investigation; writing—review and editing. **Yongjun Li**: Conceptualization; data curation; formal analysis; investigation; methodology; writing—review and editing. **Kristy Hobson**: Methodology; data curation; resources; writing—review and editing. **Muhammad Ahsan Asif**: Methodology; data curation; writing—review and editing. **Matthew Hayden**: Project Administration; resources; supervision; writing—review and editing. **Sukhjiwan Kaur**: Conceptualization; resources; supervision; writing—review and editing.

## CONFLICT OF INTEREST STATEMENT

The authors declare no conflicts of interest.

## Supporting information




**Fig. S1. BayesR3 posterior inclusion probability (PIP) for Average Internal Infection (AveIntInf) and Survival Percentage (SurPct) in canola**. The x‐axis shows the variant positions grouped by chromosome and the y‐axis shows the posterior includion probability (PIP) estimated using BayesR3. The red horizontal line indicates QTL detection threshold (PIP = 0.4), and red points indicate variants exceeding this threshold.

Supplementary Table 1. Expected heterozygosity measured using genotypes generated from each speed breeding cycle.

## Data Availability

The data that support the findings of the study “Enhanced Resistance in Chickpea and Canola via Computational‐assisted Cross Selection” will be openly available upon acceptance of the manuscript for publication on request.
