# Correction to “Enhanced disease resistance in chickpea and canola via MAGPIE: Major‐Effect Allele and Genomic Polygenic Integration for Enhancement”

**DOI:** 10.1002/tpg2.70340

**Published:** 2026-11-13

1

Lin, Z., Berg, I. V. D.,Yazdifar, S., Sudheesh, S., Riaz, A., Barbulescu, D., Li, Y., Hobson, K., Asif, M. A., Hayden, M., & Kaur, S. (2026). Enhanced disease resistance in chickpea and canola via MAGPIE: Major‐Effect Allele and Genomic Polygenic Integration for Enhancement. *The Plant Genome, 19*, e70326. https://doi.org/10.1002/tpg2.70326

Portions of Figures 1, 2, and 5 were inadvertently cut off. In addition, the header rows of Table 2 appeared incorrectly.

In the published version of Figure 1, both the *upper and lower* portions of the figure were cropped in the published version (as below).


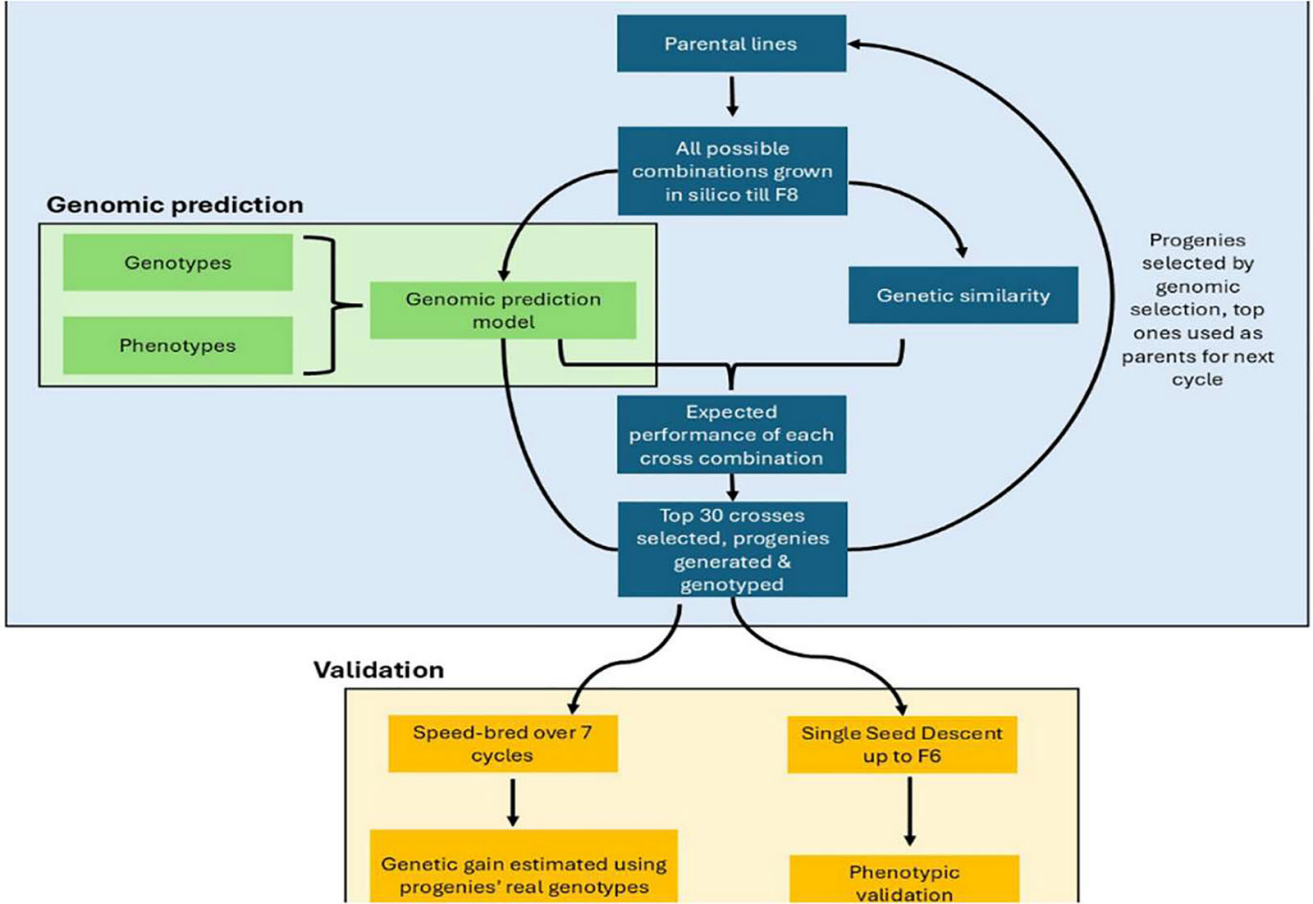


The correct Figure 1 is provided below:

**FIGURE 1:**
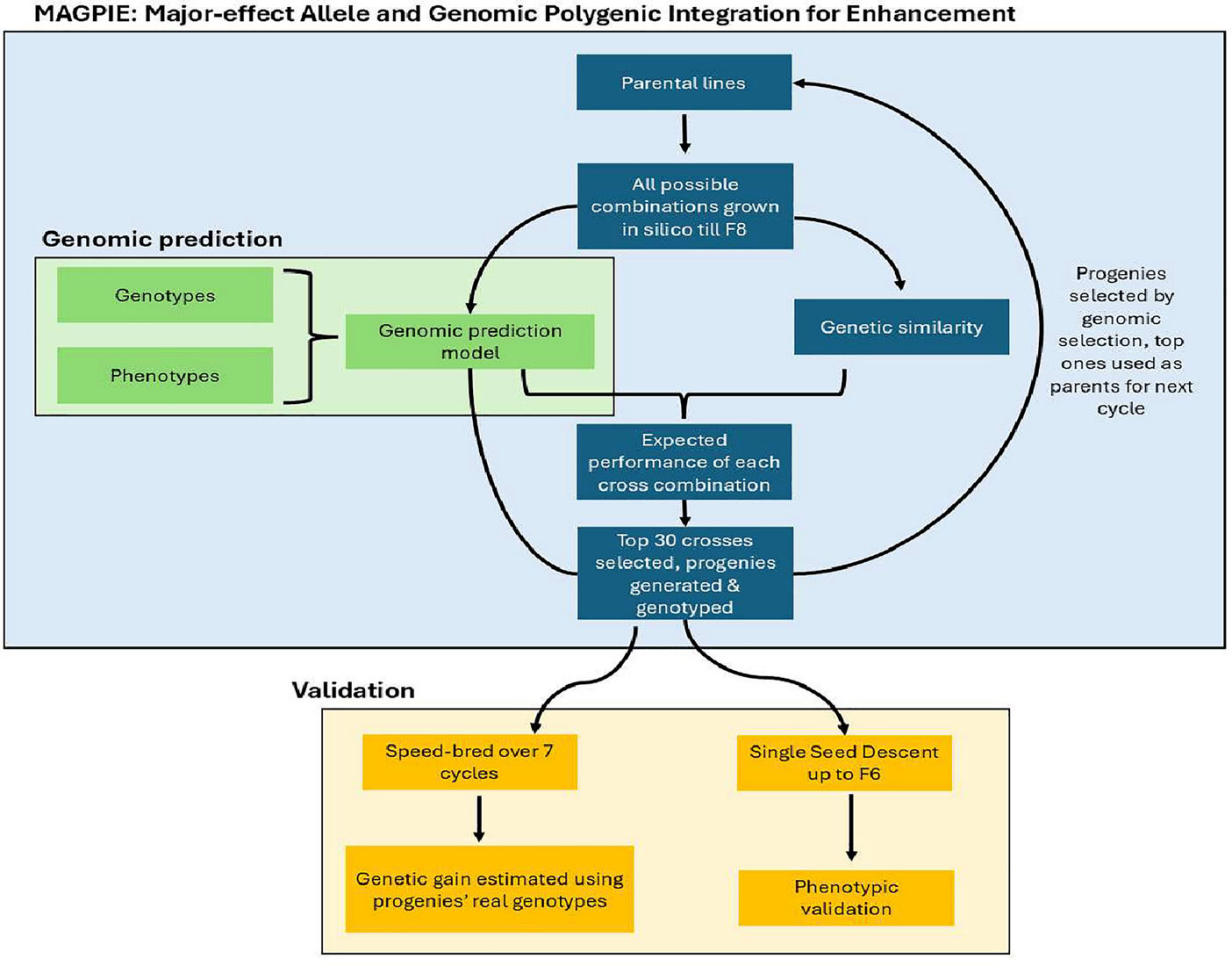
Overview of the MAGPIE (Major‐Effect Allele and Genomic Polygenic Integration for Enhancement) workflow. Phenotypic and genotypic data were used to develop genomic prediction models. These models were applied to simulated progeny from all possible parental combinations to estimate expected cross performance. Crosses predicted to generate superior progeny were selected for subsequent crossing cycles, while selected progeny were advanced through single seed descent and phenotypically validated to confirm realized genetic gain.

In the published version of Figure 2, both the *left and right* portions of the figure were cropped in the published version (as below).


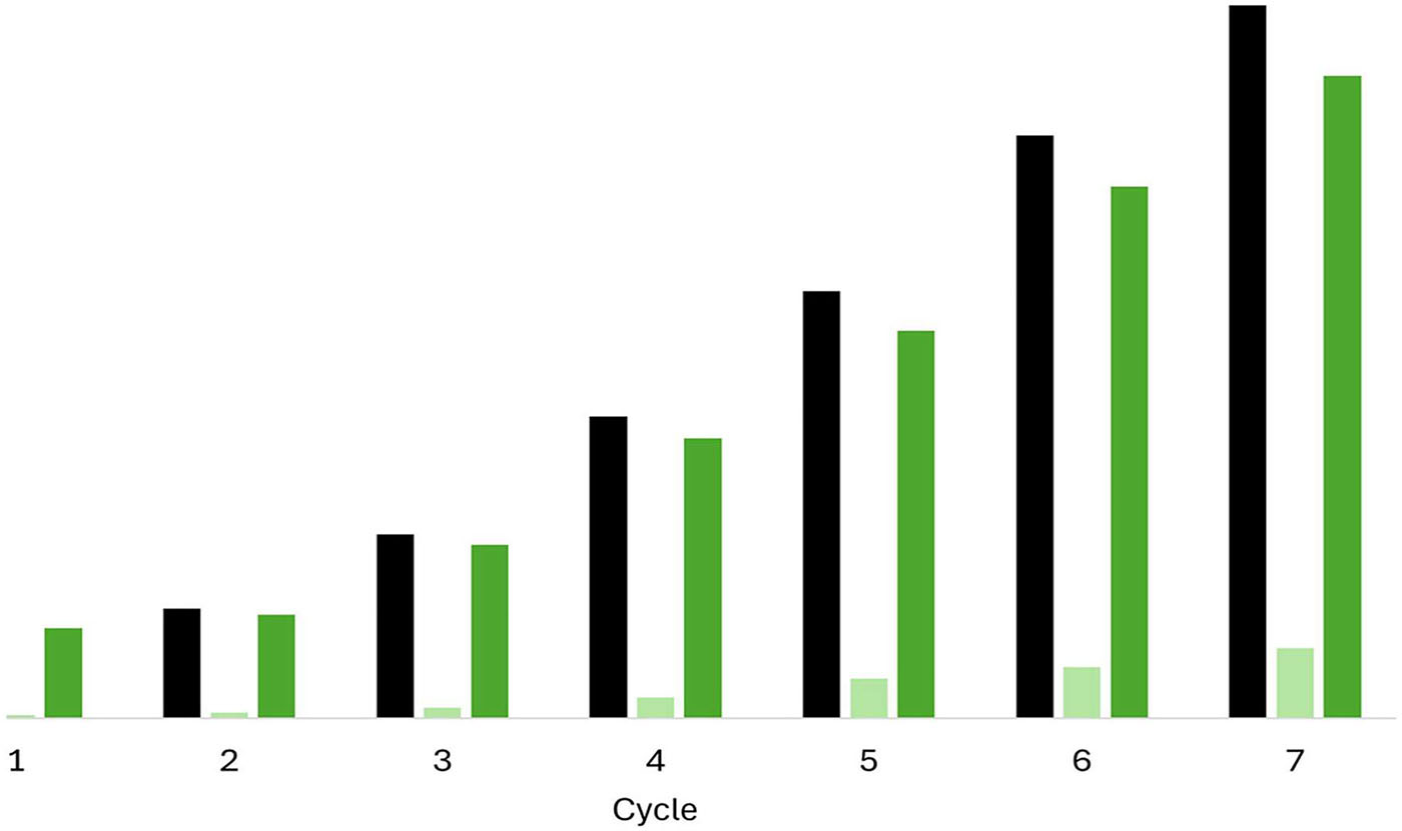


The correct Figure 2 is provided below:

**FIGURE 2:**
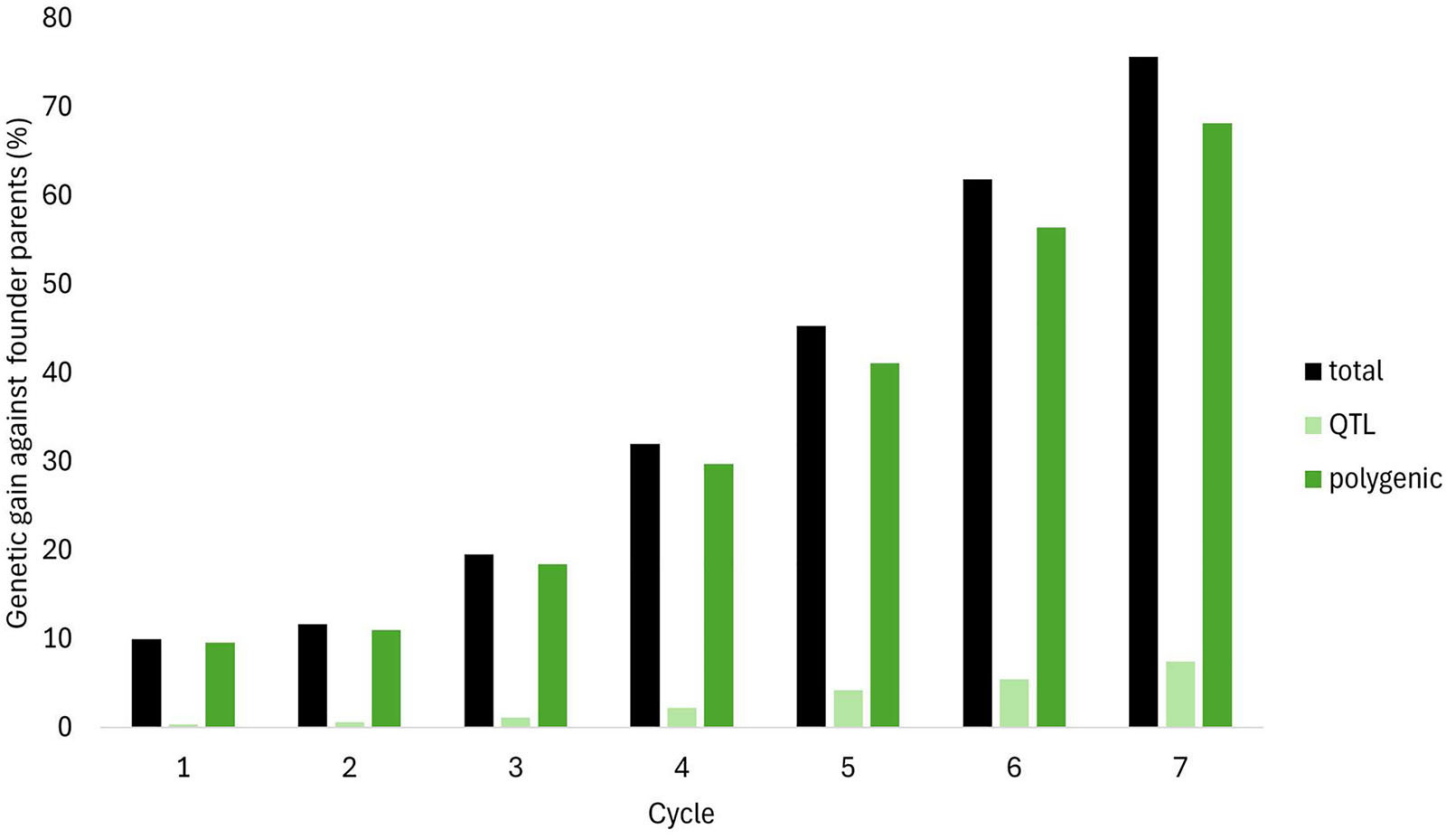
Genetic gain of Ascochyta blight resistance across seven Major‐Effect Allele and Genomic Polygenic Integration for Enhancement (MAGPIE) cycles in chickpea. The *x*‐axis shows the MAGPIE cycle, and the *y*‐axis shows the genetic gain (%) relative to the founder parents based on genomic estimated breeding values. Total genetic gain (total) was partitioned into gain attributed to major quantitative trait loci (QTL) and polygenic marker effects (polygenic). Genetic gain increased from 10% in cycle 1 to 75.7% in cycle 7, with approximately 90% of the gain attributable to polygenic effects. The QTL were identified in Lin et al. (**2025**).

In the published version of Figure 5, both the *left and right* portions of the figure were cropped in the published version (as below).


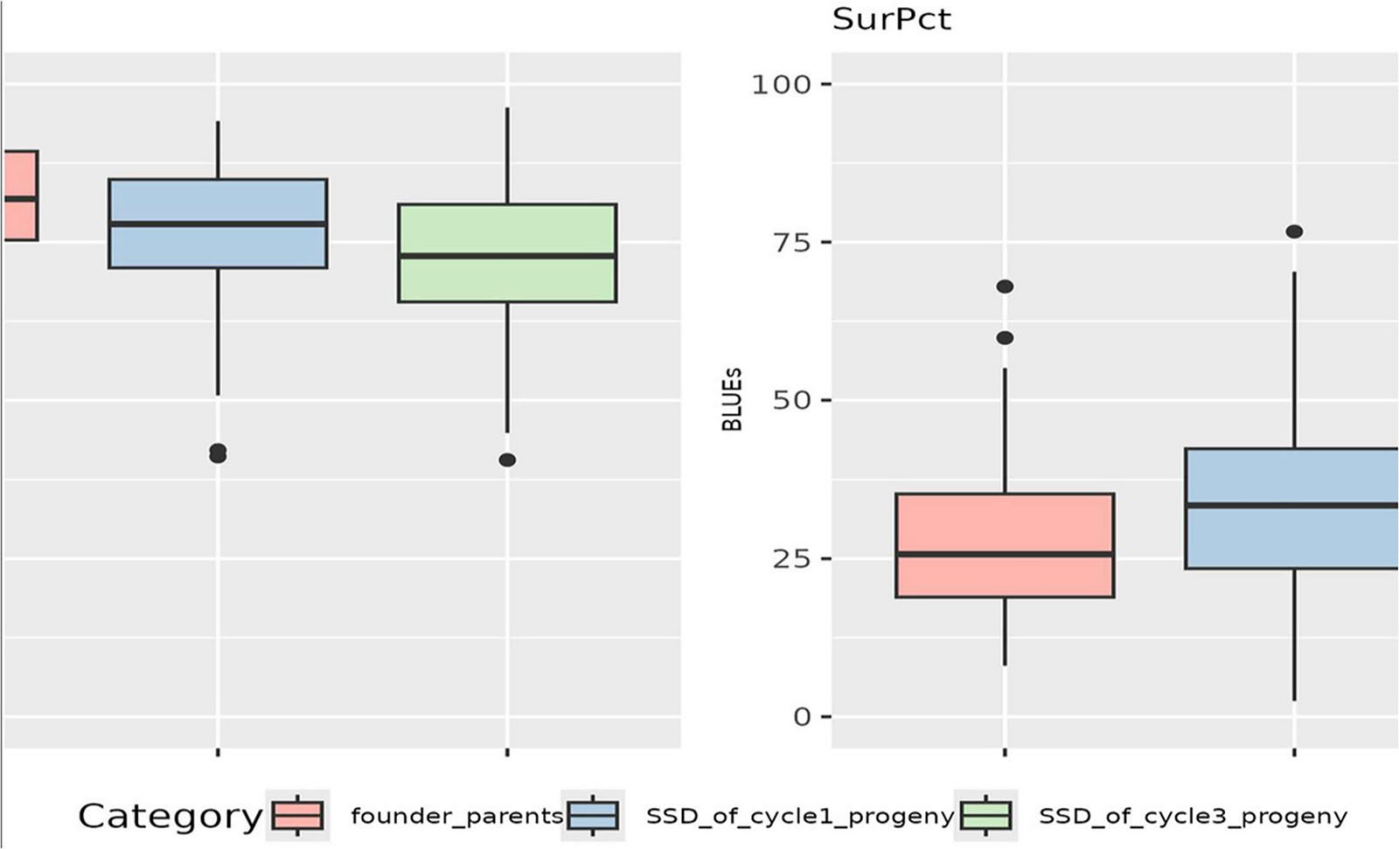


The correct Figure 5 is provided below:

**FIGURE 5:**
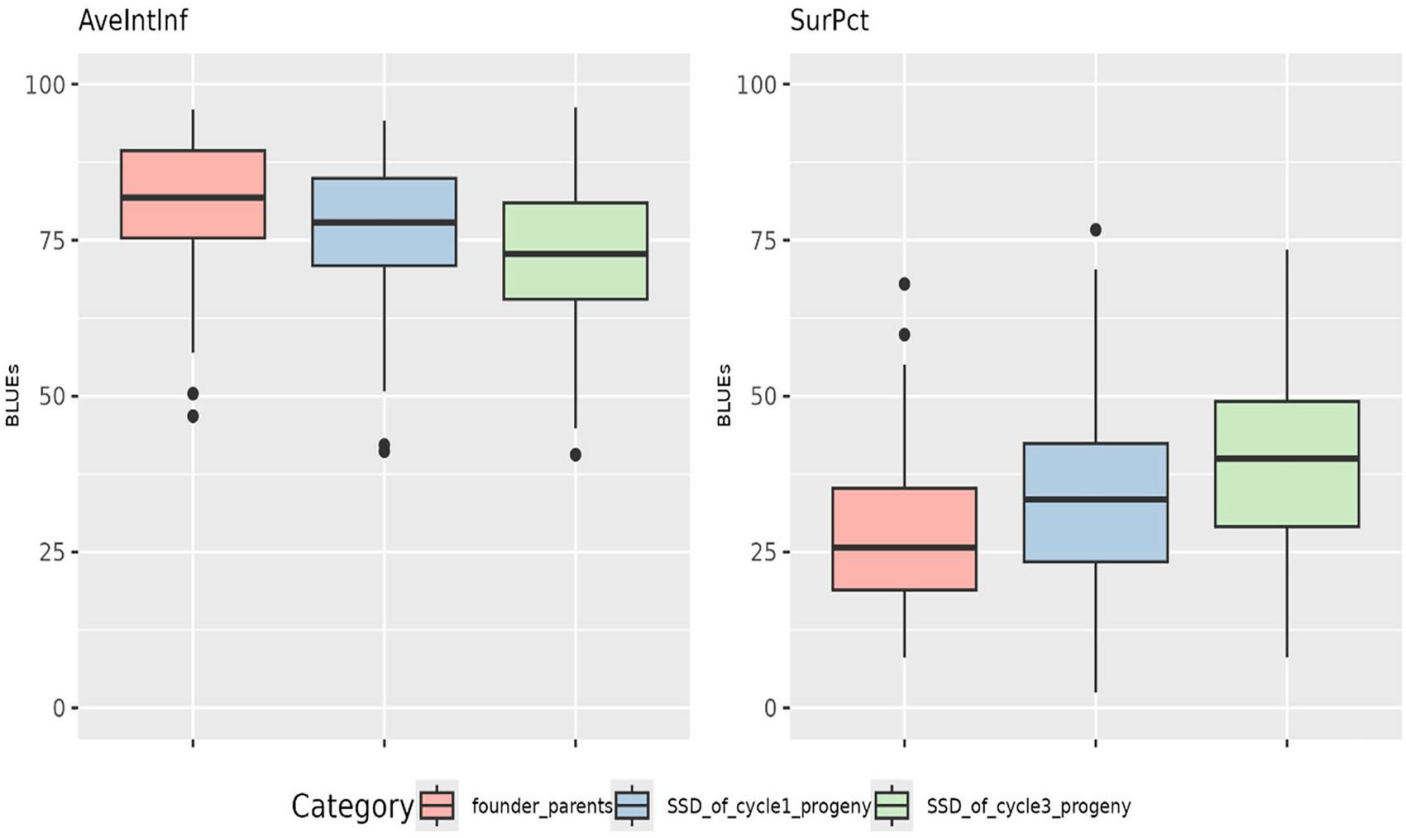
Phenotypic validation of blackleg resistance in canola founder parents and single seed descent (SSD) progeny derived from MAGPIE (Major‐Effect Allele and Genomic Polygenic Integration for Enhancement) cycles 1 and 3. Panel A shows average internal infection (AveIntInf), and panel B shows survival percentage (SurPct). The *y*‐axis represents the best linear unbiased estimates (BLUEs) for each trait. Lower average internal infection and higher survival percentage indicate greater blackleg resistance. Boxplots show the distribution of trait values for founder parents and SSD progeny from cycles 1 and 3. Compared with the founder parents, progeny from cycles 1 and 3 exhibited reduced internal infection and increased survival.

In the published version of Table 2, the header rows appeared incorrectly—vertical borders appear between the *Top 10* and *Bottom 10* sections (see red highlighted borders below).


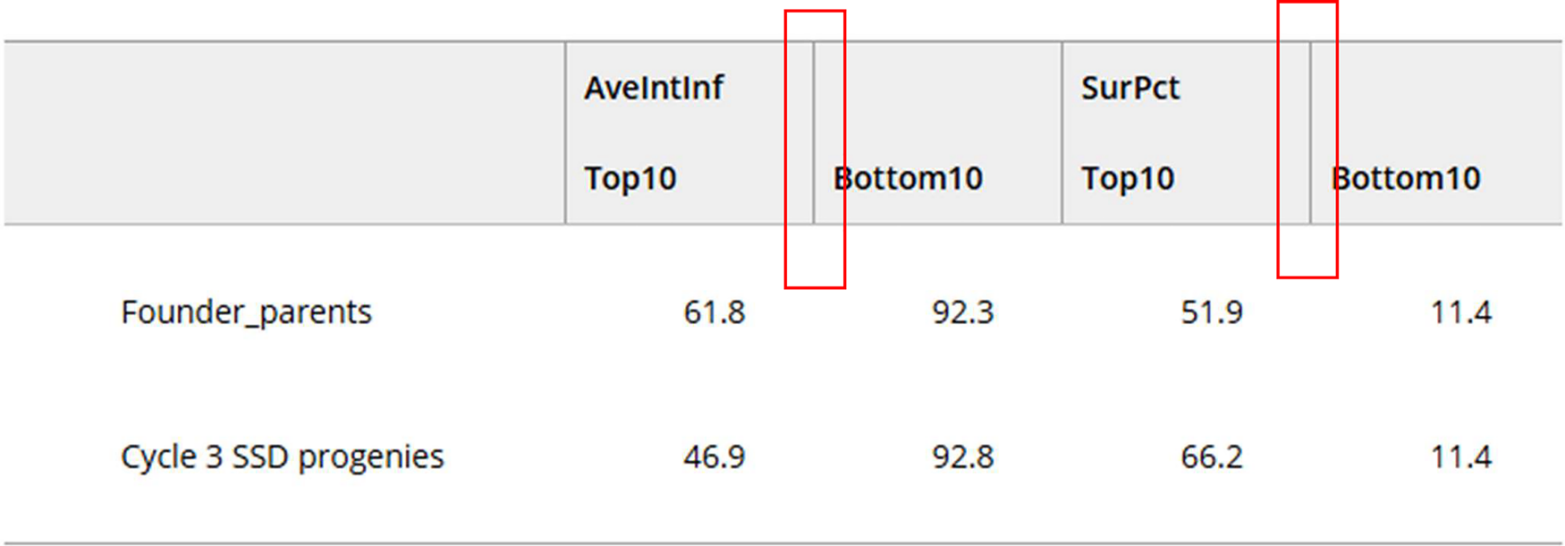


The headers have now been corrected in Table 2 below:

**TABLE 2:** Average internal infection (AveIntInf) and survival percentage (SurPct) in the 10 best (top10) and 10 worst (bottom10) performing lines in validation study. SSD, single seed descent.

	AveIntInf		SurPct	
	Top10	Bottom10	Top10	Bottom10
Founder_parents	61.8	92.3	51.9	11.4
Cycle 3 SSD progenies	46.9	92.8	66.2	11.4

We apologize for these errors.

