# Supplementary material for: Enhanced disease resistance in chickpea and canola via MAGPIE: Major‐Effect Allele and Genomic Polygenic Integration for Enhancement
Source: Plant Genome. 2026 Oct 12;19(4):e70326. doi: 10.1002/tpg2.70326 (PMC13658840; doi:10.1002/tpg2.70326)
Supplement: Supplementary file 1 [file TPG2-19-e70326-s001.pdf]

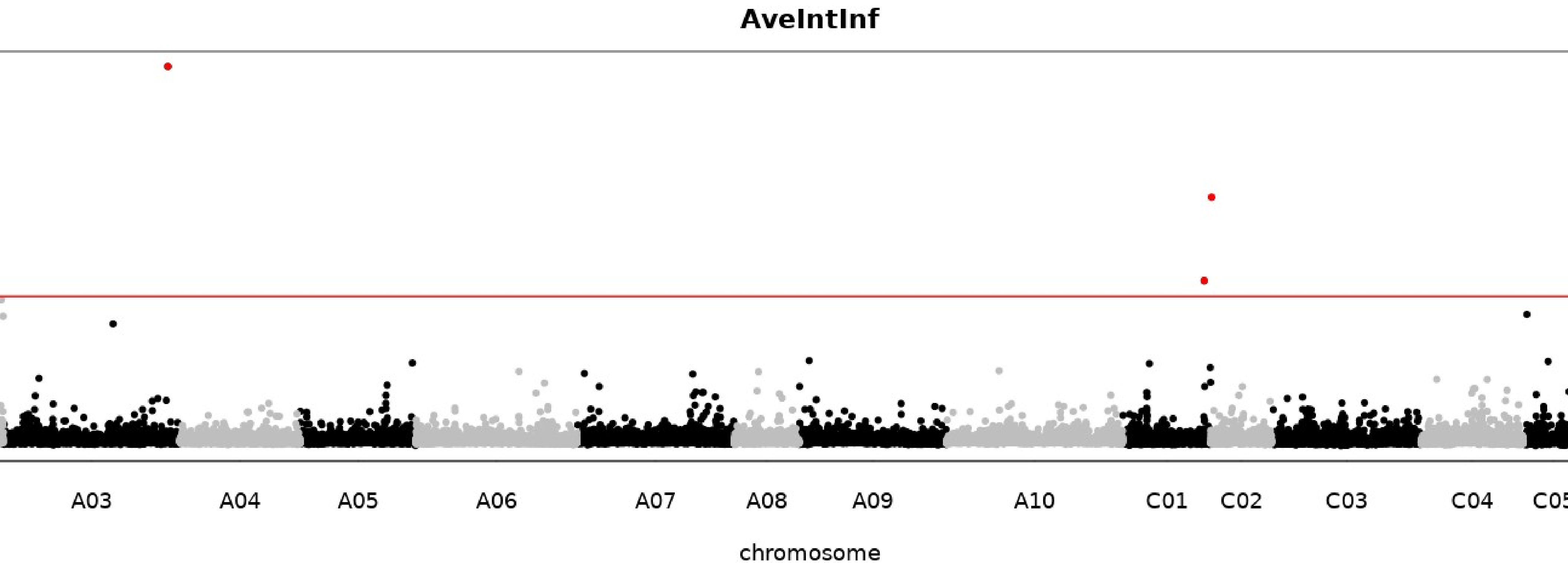
AveIntInf
A03
A04
A05
A06
A07
A08
A09
A10
C01
C02
C03
C04
C05
chromosome

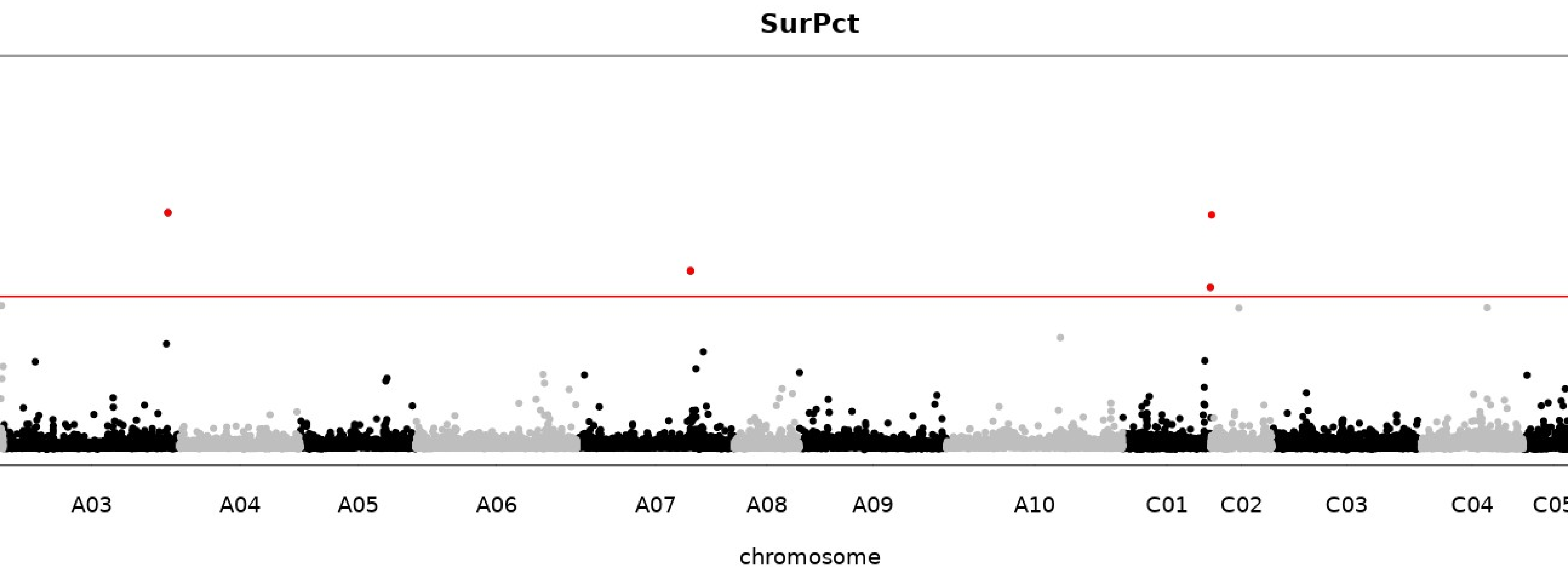
SurPct
A03
A04
A05
A06
A07
A08
A09
A10
C01
C02
C03
C04
C05
chromosome
