# Supplementary material for: Enhanced disease resistance in chickpea and canola via MAGPIE: Major‐Effect Allele and Genomic Polygenic Integration for Enhancement
Source: Plant Genome. 2026 Oct 12;19(4):e70326. doi: 10.1002/tpg2.70326 (PMC13658840; doi:10.1002/tpg2.70326)
Supplement: Supplementary file 2 [file TPG2-19-e70326-s002.docx]

|  | **Expected heterozygosity** | |
| --- | --- | --- |
| **Cycle** | **Chickpea** | **Canola** |
| **Parents** | 0.27 | 0.32 |
| **1** | 0.22 | 0.26 |
| **2** | 0.22 | 0.24 |
| **3** | 0.21 | 0.23 |
| **4** | 0.22 | 0.24 |
| **5** | 0.20 | 0.23 |
| **6** | 0.18 | 0.25 |
| **7** | 0.17 | 0.23 |

**Supplementary Table 1. Expected heterozygosity measured using genotypes generated from each speed breeding cycle.**
